# Early reapplication of prone position during venovenous ECMO for acute respiratory distress syndrome: a prospective observational study and propensity-matched analysis

**DOI:** 10.1186/s13613-024-01365-4

**Published:** 2024-08-20

**Authors:** Rui Wang, Xiao Tang, Xuyan Li, Ying Li, Yalan Liu, Ting Li, Yu Zhao, Li Wang, Haichao Li, Meng Li, Hu Li, Zhaohui Tong, Bing Sun

**Affiliations:** Department of Respiratory and Critical Care Medicine, Beijing Institute of Respiratory Medicine and Beijing Chao-Yang Hospital, Capital Medical University, No. 8 Gongren Tiyuchang Nanlu, Chaoyang District, Beijing, 100020 China

**Keywords:** Prone position, Acute respiratory distress syndrome; venovenous extracorporeal membrane oxygenation, Respiratory mechanics

## Abstract

**Background:**

A combination of prone positioning (PP) and venovenous extracorporeal membrane oxygenation (VV-ECMO) is safe, feasible, and associated with potentially improved survival for severe acute respiratory distress syndrome (ARDS). However, whether ARDS patients, especially non-COVID-19 patients, placed in PP before VV-ECMO should continue PP after a VV-ECMO connection is unknown. This study aimed to test the hypothesis that early use of PP during VV-ECMO could increase the proportion of patients successfully weaned from ECMO support in severe ARDS patients who received PP before ECMO.

**Methods:**

In this prospective observational study, patients with severe ARDS who were treated with VV-ECMO were divided into two groups: the prone group and the supine group, based on whether early PP was combined with VV-ECMO. The proportion of patients successfully weaned from VV-ECMO and 60-day mortality were analyzed before and after propensity score matching.

**Results:**

A total of 165 patients were enrolled, 50 in the prone and 115 in the supine group. Thirty-two (64%) and 61 (53%) patients were successfully weaned from ECMO in the prone and the supine groups, respectively. The proportion of patients successfully weaned from VV-ECMO in the prone group tended to be higher, albeit not statistically significant. During PP, there was a significant increase in partial pressure of arterial oxygen (PaO_2_) without a change in ventilator or ECMO settings. Tidal impedance shifted significantly to the dorsal region, and lung ultrasound scores significantly decreased in the anterior and posterior regions. Forty-five propensity score-matched patients were included in each group. In this matched sample, the prone group had a higher proportion of patients successfully weaned from VV-ECMO (64.4% vs. 42.2%; *P* = 0.035) and lower 60-day mortality (37.8% vs. 60.0%; *P* = 0.035).

**Conclusions:**

Patients with severe ARDS placed in PP before VV-ECMO should continue PP after VV-ECMO support. This approach could increase the probability of successful weaning from VV-ECMO.

**Trial Registration:**

ClinicalTrials.Gov: NCT04139733. Registered 23 October 2019.

**Supplementary Information:**

The online version contains supplementary material available at 10.1186/s13613-024-01365-4.

## Background

Prone positioning (PP) has been shown to improve outcomes for intubated patients with moderate to severe acute respiratory distress syndrome (ARDS) [1]. PP became standard care after the PROSEVA trial was published [2] and was used even more after the coronavirus disease 2019 (COVID-19) pandemic began [3]. A contemporaneous study reported that using venovenous extracorporeal membrane oxygenation (VV-ECMO) to manage severe ARDS has evolved from a salvage therapy to a more routine therapy [4]. Two randomized controlled trials and a recent systematic review and individual patient data meta-analysis consistently showed that VV-ECMO could improve the clinical outcomes of patients with severe ARDS [5–7].

While both PP and VV-ECMO have been independently shown to improve ARDS patient outcomes, combining both therapies has a sound rationale. Several recent observational studies and a meta-analysis demonstrated that PP during VV-ECMO may reduce mortality [8–12]. In terms of timing, early initiation of PP after VV-ECMO has a more favorable prognosis than late PP [13, 14]. This difference in outcome may be attributed to the potential development of mechanical ventilation-induced pulmonary fibrosis, which could diminish the benefits of PP [15, 16]. It should be noted that more than half of patients in the EOLIA trial received PP before VV-ECMO [6], which indicates that PP is not practical for all patients, and for some of them, a further worsening of respiratory failure or the impossibility of maintaining protective ventilation may require VV-ECMO. It is unclear whether this subset of patients should be placed in the prone position after receiving VV-ECMO. A recently published PRONECMO study partially addressed this question. The study, which involved mainly COVID-19-related ARDS patients who had all nearly undergone PP before ECMO, did not find benefits from routine PP after ECMO [17].

To further address this question, we hypothesized that early use of PP during VV-ECMO, despite the limited efficacy before VV-ECMO, may be associated with better outcomes in patients with severe ARDS, especially non-COVID-19 patients. Therefore, we conducted a prospective observational study to determine whether early use of PP during VV-ECMO would increase the proportion of patients successfully weaned from VV-ECMO compared with supine positioning in patients who received PP before ECMO.

## Methods

### Study design and patients

This was a prospective observational study (ClinicalTrials.gov, NCT04139733). Patients were recruited from the respiratory intensive care unit (ICU) of Beijing Chao-Yang Hospital. The study was approved by the Research Ethics Board (REB) of the Affiliated Beijing Chao-Yang Hospital, Capital Medical University (protocol number: 2019-KE-171; protocol title: “Early Use of Prone Position in ECMO for Severe ARDS”; approval date: September 30, 2019). All procedures were followed according to the ethical standards of the REB of the Affiliated Beijing Chao-Yang Hospital, Capital Medical University and the Helsinki Declaration of 1975. Since patients were unable to provide written consent at the time of inclusion, written informed consent was obtained from their legal guardians.

All patients included in our study met the Berlin definition of ARDS and underwent PP before VV-ECMO [18]. VV-ECMO was implemented for patients who met any of the following criteria: Despite optimum mechanical ventilation (tidal volume 6 ml/kg of predicted body weight [PBM], positive end-expiratory pressure [PEEP] ≥ 10 cmH_2_O, fraction of inspired oxygen [FiO_2_] ≥ 0.8), and use of rescue therapies such as PP: (1) ratio of partial pressure of arterial oxygen (PaO_2_) to FiO_2_ < 50 mm Hg for > 3 h; (2) PaO_2_/FiO_2_ < 80 mm Hg for > 6 h; (3) arterial blood pH < 7.25 with partial pressure of arterial carbon dioxide (PaCO_2_) ≥ 60 mmHg for > 6 h, with respiratory rate increased to 35 breaths/min and mechanical ventilation settings adjusted to maintain plateau pressure (P_plat_) ≤ 32 cm H_2_O [6].

Patients were divided into the prone and supine groups according to whether early PP was combined with VV-ECMO.

### Prone positioning

In our respiratory ICU, PP is routinely performed during ECMO, with the final decision left to treating physicians. In the prone group, patients started PP within 24 h after initiating VV-ECMO treatment. A single PP session lasts 16 h continuously for at least 5 days, unless interrupted for life-threatening complications. Patients were kept under deep sedation during PP sessions. Procedures and healthcare workers’ positions for performing PP are shown in the online supplemental methods Section S1 and Figure S1.

Patients with any of the following conditions were not to be placed in PP: (1) facial or neck trauma; (2) spinal instability; (3) recent thoracic surgery; (4) elevated intracranial pressure; (5) hemoptysis; or (6) hemodynamic instability (i.e., mean arterial pressure [MAP] < 65 mm Hg and norepinephrine > 0.5 ug/kg/min).

The criteria for stopping PP were any of the following: (1) greater than 1 L/min increase of ECMO blood flow to achieve saturation of pulse oxygen (SpO_2_) ≥ 92%; (2) norepinephrine > 0.5 ug/kg/min to maintain MAP ≥ 65 mm Hg; (3) frequent abrupt declines in blood flow; or (4) any other life-threatening reason for which the physician decided to stop the treatment.

### VV-ECMO management

VV-ECMO cannulas were inserted by trained intensivists with femoral-jugular access as the primary choice. The VV-ECMO blood flow was regulated to maintain SpO_2_ > 92%. After the initiation of ECMO, the sweep gas is initially set at 2 L/min and the flow rate is gradually increased to achieve PaCO_2_ level below 45 mm Hg. We used a transpulmonary pressure (P_tp_)-guided ventilation approach during VV-ECMO for all patients included in the study (online supplemental methods Section S2). Ventilator settings were pressure-assist control mode, inspiratory pressure was lowered to keep peak airway pressure (P_peak_) < 25 cm H_2_O, PEEP was set at such a level that expiratory P_tp_ stayed between 0 and 5 cm H_2_O, respiratory rate was 10 breaths/min, and FiO_2_ was < 0.5 [19]. Systemic anticoagulation with unfractionated heparin was required to maintain an activated partial thromboplastin time (APTT) of 50 to 70 s.

Initially, patients were sedated with propofol, midazolam, and remifentanil to achieve a Richmond Agitation and Sedation Scale (RASS) between − 5 and − 3. Neuromuscular blockade was not routinely used unless in cases of strong respiratory drive and concerns about self-inflicted lung injury [20]. After a patient was stable for 24 h on VV-ECMO, the RASS goal was lowered to -2-0 until decannulation. The patient was weaned from VV-ECMO after successful treatment of the underlying disease and improved native lung function. A weaning test was attempted by stopping the gas flow for 2 h. The device was withdrawn if the arterial blood gas demonstrated PaO_2_ ≥ 70 mm Hg, PaCO_2_ < 50 mm Hg, stable hemodynamics, and ventilator settings to allow protective ventilation (i.e., tidal volume 6 ml/kg of PBM, P_plat_ ≤30 cm H_2_O, PEEP < 12 cm H_2_O, respiratory rate 10 to 30 breaths/min and FiO_2_ ≤ 0.6).

### EIT monitoring

Electrical impedance tomography (EIT) data were acquired using a standard device (PulmoVista^®^500; Dräger, Lübeck, Germany) with a sample rate of 20 Hz. The EIT belt was positioned directly below the armpits, between the fourth and fifth intercostal spaces. The belt was kept in the same position during both supine and prone positioning. EIT data were generated by applying a small alternating electrical current. For each patient, we used the same baseline reference. EIT data were continuously visualized on the PulmoVista screen during PP without modification of the belt. EIT images were continuously recorded at 20 Hz for 5 min at four different time points.

The data were digitally filtered using a low-pass filter with a cut-off frequency of 40/min to eliminate small impedance changes synchronous with the heart rate. Lung images were divided into four non-overlapping ventral-to-dorsal horizontal regions of interest (ROIs): ventral, medial-ventral, medial-dorsal, and dorsal. The vertical height of these ROIs was identical and corresponded to 25% of the anteroposterior diameter. The EIT scans consisted of images showing impedance with 32 × 32 color-coded matrices. Output pixel values represented changes in local impedance, between the end of expiratory and inspiratory periods.

### Lung ultrasound

Lung ultrasound was performed by two experienced sonographers using a Philips CX50 portable ultrasound system (Philips Healthcare, Andover, MA, USA) equipped with convex (1–5 MHz) probes. The surface anatomy of the lung lobes and lung ultrasound zones are shown in Figure S2. Each lung was divided into anterior, lateral, and posterior regions by the parasternal line, anterior axillary line, posterior axillary line, and paravertebral lines. The anterior region was further equally divided into four areas by the clavicular midline and horizontal line. The lateral region was divided into upper and lower zones by the horizontal line. The parascapular line divided the posterior region into two unequal areas, then every area was divided into three areas by two horizontal lines for five examination areas (the scapula covers area was ruled out). Therefore, there were 11 examination points for a single lung and 22 for both lungs.

The sonographic signs of lung aeration were classified into four categories: (1) Score 0: A-line or two or fewer well-spaced B-lines; (2) Score 1: Three or more well-spaced B-lines; (3) Score 2: Coalescent B-lines; or (4) Score 3: Tissue-like pattern [21]. Lung ultrasound scores were calculated as the sum of points at each region.

### Endpoints and measurements

The primary endpoint was the proportion of patients successfully weaned from VV-ECMO, which meant weaning after more than 48 h with stable oxygenation and no need to re-establish ECMO. The secondary endpoint was mortality at 60 days.

In both groups, we collected the following parameters before starting ECMO: age, sex, body mass index, etiology of pneumonia, underlying comorbidities, days of mechanical ventilation before ECMO, rescue therapy, pre-ECMO scores, hemodynamic status, arterial blood gases, and ventilatory variables.

For the first PP cycle, the following four different time points were identified: (1) supine before PP (1 h before PP); (2) during PP (4 h after PP); (3) end PP (end of PP period); and (4) Supine after PP (1 h after supination). At each time point, ventilatory variables, ECMO setting, arterial blood gas, EIT data, and lung ultrasound scores were collected. In the prone group, we also collected the following: ECMO hours before pronation, duration and number of PP sessions, and complications of PP maneuvers during VV-ECMO support. Lastly, we recorded other endpoints for each group, including successfully weaned from VV-ECMO for 30 days (defined as a patient survived without ECMO or lung transplant for 30 days after ECMO discontinuation), length of ICU and hospital stay, ECMO duration, ventilator-free days at day 60, combined renal replacement therapy (CRRT), tracheostomy, and ECMO-related complications.

### Statistical analysis

Results for continuous variables are given as either means (± standard deviation) or medians (with interquartile ranges). Groups were compared using either Student’s t-test or the Mann-Whitney U test, as appropriate. For categorical variables, the percentages of patients in each category were compared using a chi-square test or Fisher’s exact test. Variables from the first PP session during VV-ECMO were compared using two-way analysis of variance for repeated measures. The mortality rate was compared using a Kaplan-Meier estimate of survival and a log-rank test was used to compare the two groups There was no imputation for missing data (Table S1).

*Propensity Score.* Covariates presumed to be associated with PP during VV-ECMO, successful weaning from VV-ECMO, and 60-day survival status were included in a multivariable logistic regression analysis with PP as the dependent variable to determine each patient’s propensity score (PS) for PP during VV-ECMO. We included in this model the same set of clinically meaningful variables selected for a previous study [22]: age, body mass index, sequential organ failure assessment (SOFA) score, duration of mechanical ventilation before VV-ECMO, duration and number of PP sessions, and PaO_2_/FiO_2_ before VV-ECMO. Immunocompromised patients had significantly higher ICU and hospital mortality despite similar ARDS severity [23]. Therefore, immunocompromised status was also added to PS.

*Case-Matching Procedure.* Patients in the prone and supine group were matched according to their PS using 1:1 matching without replacement and a 0.2 caliper width. Covariate balance between the two groups was assessed after matching, and we considered an absolute standardized mean difference less than 0.1 as evidence of balance. The dot-plot of covariates included in the PS (Figure S3) showed that the two groups were comparable.

All *P* values were two-sided, and values less than 0.05 were considered significant. Data were analyzed using SPSS version 22.0 (IBM Corp., Armonk, NY, USA) and GraphPad Prism 8 (GraphPad Software Inc., San Diego, CA, USA).

## Results

### Study population

From November 2019 to August 2023, 165 patients with severe ARDS supported by VV-ECMO were enrolled, with 50 patients in the prone group and 115 patients in the supine group (Figure S4). The characteristics of the study population are summarized in Table 1. Briefly, the primary cause of ARDS was viral pneumonia, accounting for 64% of cases, with 13 cases (8%) attributed to COVID-19 pneumonia. There was no significant difference between the two groups. The median time between mechanical ventilation and VV-ECMO initiation was 1 (range, 1–4) days. Before VV-ECMO, the duration of PP was 16 (range, 14–18) hours, and the number of PP sessions was 1 (range, 1–4) per patient.

**Table 1:** Demographic characteristics, pre-ECMO treatments, hemodynamic status, arterial blood gas and ventilatory variables

Characteristic	All patients (*n* = 165)	Prone group (*n* = 50)	Supine group (*n* = 115)	*P*
Age (years)	56.0 ± 15.7	58.0 ± 13.7	55.1 ± 16.6	0.281
Male, no. (%)	113 (68.5)	38 (76.0)	75 (65.2)	0.171
Body mass index (kg/m^2^)	24.5 (22.5–26.3)	25.0 (22.9–26.4)	24.2 (22.4–26.2)	0.448
Pulmonary pathogen spectrum, no. (%)				
Bacterial	37 (22.4)	12 (24.0)	25 (21.7)	0.749
Viral	106 (64.2)	30 (60.0)	76 (66.1)	0.453
COVID-19 pneumonia	13 (7.9)	4 (8.0)	9 (7.8)	1.000
Fungal	3 (1.8)	1 (2.0)	2 (1.7)	1.000
Pneumocystis jiroveci	19 (11.5)	7 (14.0)	12 (10.4)	0.510
Comorbidity, no. (%)				
Immunocompromised	29 (17.6)	11 (22.0)	18 (15.7)	0.325
Coronary artery disease	23 (13.9)	6 (12.0)	17 (14.8)	0.635
Hypertension	59 (35.8)	22 (44.0)	37 (32.2)	0.145
Diabetes mellitus	24 (14.5)	9 (18.0)	15 (13.0)	0.407
Chronic renal insufficiency	9 (5.5)	3 (6.0)	6 (5.2)	1.000
Pre-ECMO Scores				
Murray score	3.50 (3.25–3.75)	3.50 (3.50–3.75)	3.50 (3.25–3.75)	0.258
SOFA score	13 (11–14)	12 (11–14)	13 (11–14)	0.213
APACHE II score	17 (14–21)	17 (14–24)	18 (15–21)	0.680
RESP score	2 (0–3)	2 (0–3)	2 (1–3)	0.436
Pre-ECMO variables				
Days of MV before ECMO (days)	1 (1–4)	2 (1–3)	1 (1–4)	0.213
Rescue therapy, no. (%)				
Corticosteroids	41 (24.8)	11 (22.0)	30 (26.1)	0.577
Prone positioning	165 (100.0)	50 (100.0)	115 (100.0)	1.000
Duration of PP (hours/day)	16 (14–18)	16 (13–17)	16 (14–18)	0.182
Number of PP sessions	1 (1–4)	2 (1–3)	1 (1–4)	0.477
Lung recruitment maneuvers	87 (52.7)	23 (46.0)	64 (55.7)	0.254
Neuromuscular blockade	22 (13.3)	7 (14.0)	15 (13.0)	0.868
HFOV	6 (3.6)	2 (4.0)	4 (3.5)	1.000
Inhaled nitric oxide	9 (5.5)	3 (6.0)	6 (5.2)	0.889
Hemodynamic status				
Vasopressor, no. (%)	111 (67.3)	36 (72.0)	75 (65.2)	0.393
Lactate (mmol/L)	1.7 (1.4–2.5)	1.7 (1.2–2.7)	1.7 (1.4–2.4)	0.886
Heart rate (beats/min)	108.4 ± 23.1	104.5 ± 25.6	110.1 ± 21.9	0.151
Mean arterial pressure (mmHg)	76.7 ± 13.9	78.4 ± 12.8	76.0 ± 14.4	0.313
Arterial blood gas				
pH	7.36 (7.29–7.43)	7.36 (7.32–7.43)	7.37 (7.28–7.43)	0.521
PaO_2_ (mmHg)	59.1 ± 12.5	59.3 ± 10.6	59.1 ± 13.2	0.921
PaCO_2_ (mmHg)	47.4 ± 12.5	46.6 ± 12.3	47.8 ± 12.6	0.574
HCO_3_^−^ (mmol/L)	25.6 ± 5.3	25.1 ± 5.9	25.8 ± 5.0	0.503
SaO_2_ (%)	89 (84–92)	89 (86–91)	89 (84–94)	0.552
PaO_2_:FiO_2_ ratio (mmHg)	59.5 ± 12.8	59.5 ± 10.5	59.6 ± 13.8	0.996
Ventilatory variables				
PEEP (mmH_2_O)	14.3 ± 3.6	14.7 ± 2.9	14.2 ± 3.9	0.354
Tidal volume (ml)	393 ± 52	401 ± 59	389 ± 48	0.158
Tidal volume (ml/PBM)	6.0 ± 0.6	6.0 ± 0.9	6.0 ± 0.4	0.859
Respiratory rate (breaths/min)	28.7 ± 5.4	28.4 ± 4.0	28.8 ± 5.9	0.641
Plateau pressure (cmH_2_O)	28.4 ± 4.3	28.1 ± 3.9	28.5 ± 4.5	0.635
Peak airway pressure (cmH_2_O)	32.3 ± 4.3	31.8 ± 3.1	32.4 ± 4.8	0.305
Driving pressure (cmH_2_O)	14.4 ± 3.6	14.5 ± 4.1	14.3 ± 3.3	0.788
Compliance (ml/cmH_2_O)	25.6 ± 8.1	25.9 ± 6.9	25.4 ± 8.6	0.674

COVID-19 coronavirus disease 2019, SOFA sequential organ failure assessment, APACHE II Acute Physiology and Chronic Health Evaluation II, ECMO extracorporeal membrane oxygenation, RESP Respiratory ECMO Survival Prediction, MV mechanical ventilation, HFOV high frequency oscillatory ventilation, PaO_2_ partial pressure of arterial oxygen, PaCO_2_ partial pressure of arterial carbon dioxide, HCO_3_^−^ bicarbonate, SaO_2_ arterial oxygen saturation, PaO_2_:FiO_2_ ratio of the partial pressure of arterial oxygen to the fraction of inspired oxygen, PEEP positive end-expiratory pressure, PBM predicted body weight

### Outcomes

Clinical outcomes of patients are shown in Table 2. The proportion of patients successfully weaned from VV-ECMO (32 of 50 vs. 61 of 115, *P* = 0.192) and alive (30 of 50 vs. 58 of 115, *P* = 0.258) in the prone group tended to be higher than that of the supine group, albeit not statistically significant. Kaplan-Meier analysis also indicated that the 60-day survival rate did not differ significantly between the groups (*P* = 0.279) (Figure S5). ECMO duration was significantly shorter (*P* = 0.009) in the prone group. There was no significant difference in length of ICU or hospital stay, CRRT, tracheostomy or, ECMO-related complications.

**Table 2:** Outcomes and VV-ECMO related complications

Outcome	All patients (*n* = 165)	Prone group (*n* = 50)	Supine group (*n* = 115)	*P*
Primary end point				
Proportion of patients successfully weaned from VV-ECMO no. (%)	93 (56.4)	32 (64.0)	61 (53.0)	0.192
Secondary end point				
60-day mortality no. (%)	77 (46.7)	20 (40.0)	57 (49.6)	0.258
Other end points				
Successfully weaned from VV-ECMO for 30 days	88 (53.3)	30 (60.0)	58 (50.4)	0.258
ICU length of stay (days)	28 (20–43)	28 (21–39)	28 (19–44)	0.837
Hospital length of stay (days)	33 (23–46)	35 (27–46)	30 (22–46)	0.229
ECMO duration (days)	14 (8–19)	12 (8–17)	16 (11–25)	0.009
Ventilator-free days at day 60 (days)	0 (0–38)	21 (0–41)	0 (0–38)	0.360
CRRT no. (%)	81 (49.1)	24 (48.0)	57 (49.6)	0.893
Tracheostomy no. (%)	49 (29.7)	18 (36.0)	31 (27.0)	0.243
Complications no. (%)				
ECMO mechanical complications				
Oxygenator failure	10 (6.1)	4 (8.0)	6 (5.2)	0.739
Oxygenator thrombosis	38 (23.0)	13 (26.0)	25 (21.7)	0.550
Other circuit thrombosis	5 (3.0)	2 (4.0)	3 (2.6)	1.000
Bleeding				
Gastrointestinal hemorrhage	72 (43.6)	22 (44.0)	50 (43.5)	0.950
Intracranial hemorrhage	4 (2.4)	1 (2.0)	3 (2.6)	1.000
Pulmonary hemorrhage	32 (19.4)	7 (14.0)	25 (21.7)	0.248
Cannulation-site bleeding	44 (26.7)	15 (30.0)	29 (25.2)	0.523
Other sites hemorrhage	38 (23.0)	9 (18.0)	29 (25.2)	0.312
Culture-confirmed infection				
CRBSI	10 (6.1)	3 (6.0)	7 (6.1)	1.000
VAP	50 (30.3)	15(30.0)	35 (30.4)	0.955
Urinary infection	13 (7.9)	3 (6.0)	10 (8.7)	0.782
Other sources	7 (4.2)	2 (4.0)	5 (4.3)	1.000
Barotrauma	11 (6.7)	3 (6.0)	8 (7.0)	1.000

VV-ECMO venovenous extracorporeal membrane oxygenation, ICU intensive care unit, CRRT continuous renal replacement therapy, CRBSI catheter-related blood stream infection, VAP ventilator associated pneumonia

Table S2 in the online supplement shows the univariate logistic analysis for patients successfully weaned from VV-ECMO. Covariates significantly associated with successful weaning were age, immunocompromised status, hours of mechanical ventilation before ECMO, and duration and number of PP before VV-ECMO.

### PP after VV-ECMO and complications

Median ECMO duration before the first PP was 18 (range, 14–22) hours (Table 3). The duration of PP was 15 (range, 14–16) hours, and the number of PP sessions was 5 (range, 5–6) per patient. Complications were reported during 23 (9.1%) PP sessions. Ten (4.0%) PP sessions were aborted due to complications. We did not record any accidental extubation or ECMO cannula dislodgement. Cannula-site bleeding occurred in 5 (10.0%) patients and was the most frequent complication. Other complications were airway dislodgement (8.0%), endotracheal tube obstruction (8.0%), drop in ECMO blood flow (6.0%), hemodynamic instability (6.0%), vomiting (4.0%), and facial swelling (4.0%).

**Table 3:** Prone positioning details and related complications during ECMO support

Characteristic	Prone group (*n* = 50)
Prone positioning	
ECMO hours before pronation (hours)	18 (14–22)
Duration of PP (hours/day)	15 (14–16)
Number of PP sessions	5 (5–6)
Complications no. (%)	
Major complications	
Accidental extubation	0 (0.0)
ECMO cannula dislodgment	0 (0.0)
Airway dislodgment	4 (8.0)
Endotracheal tube obstruction	4 (8.0)
Minor complications	
Drop in ECMO blood flow	3 (6.0)
Cannula-site bleeding	5 (10.0)
Hemodynamic instability	3 (6.0)
Vomiting	2 (4.0)
Facial swelling	2 (4.0)

ECMO venovenous extracorporeal membrane oxygenation

### Physiologic effects of the first PP session during VV-ECMO

**Fig. 1:**
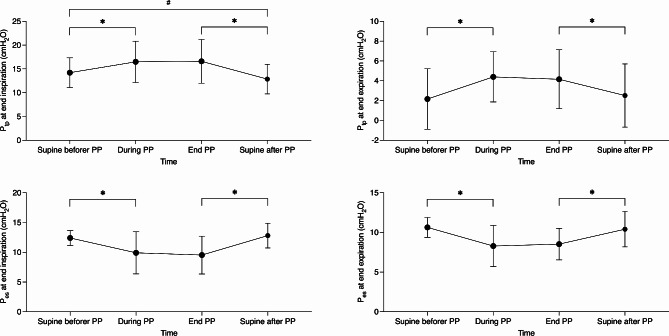
Changes in P_tp_ and P_es_ across the first PP session after VV-ECMO support. P_tp_ transpulmonary pressure, P_es_ esophageal pressure, PP prone position, VV-ECMO venovenous extracorporeal membrane oxygenation. # *P <* 0.05, * *P <* 0.01

**Fig. 2:**
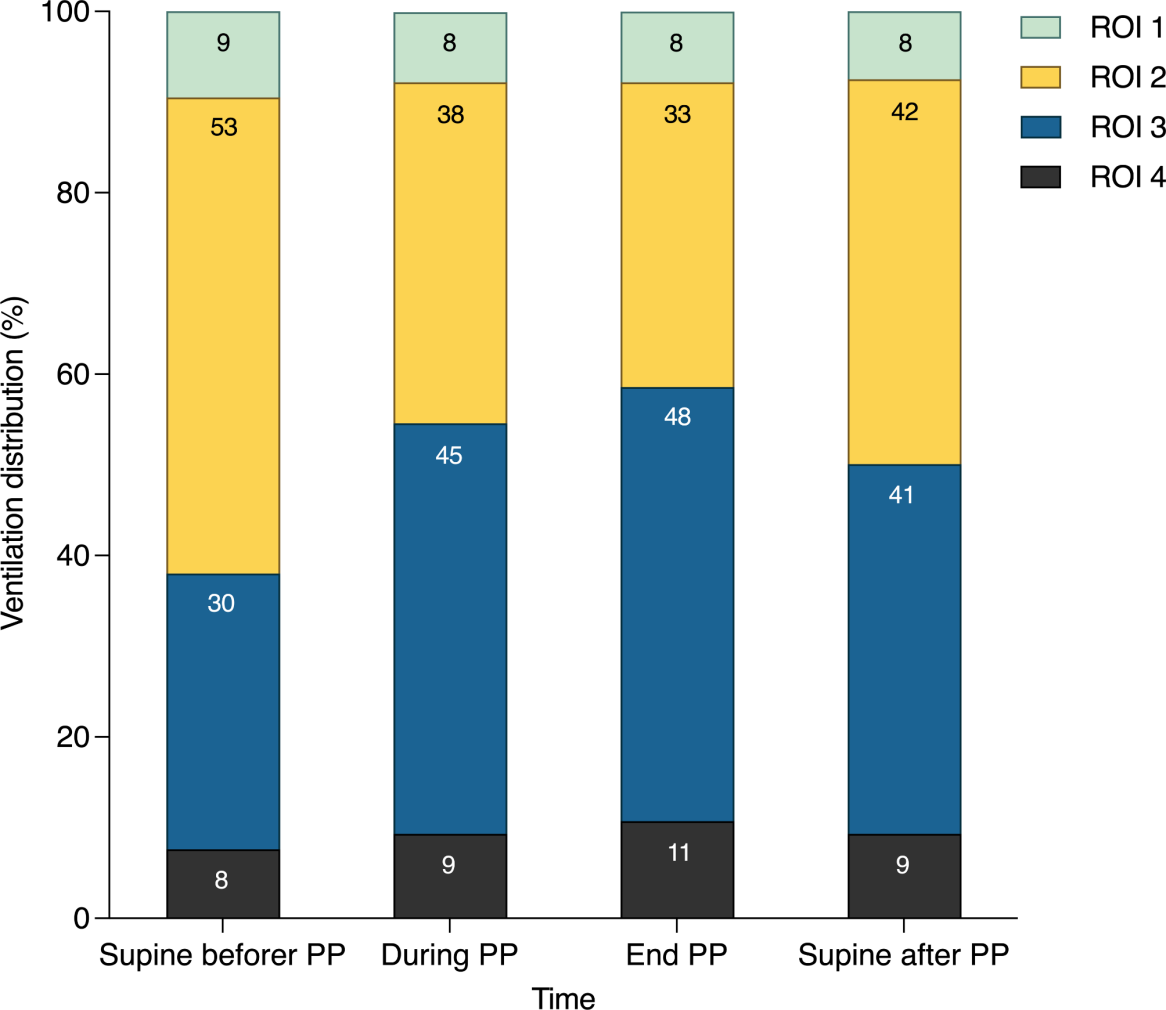
Changes in ventilation distribution at four ventral-to-dorsal horizontal regions across the first PP session after VV-ECMO support. PP prone position, VV-ECMO venovenous extracorporeal membrane oxygenation, ROI region of interest

**Fig. 3:**
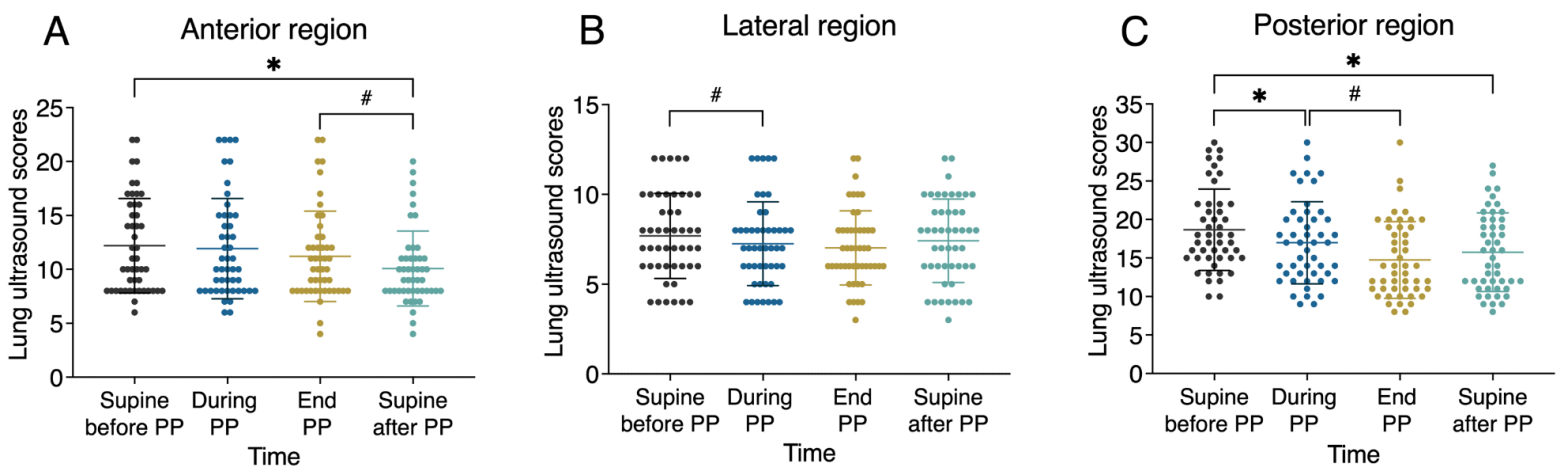
Changes in lung ultrasound scores at the anterior (**A**), lateral (**B**), and posterior (**C**) regions across the first PP session after VV-ECMO support. PP prone position, VV-ECMO venovenous extracorporeal membrane oxygenation

Physiologic parameters during the first PP are presented in Table 4. After PP, P_tp_ at end-inspiration was significantly lower than before PP (*P* = 0.037) (Fig. 1). During PP without a change in ventilator and ECMO settings, there was a significant increase in PaO_2_ (*P* < 0.001) and a slight increase in PaCO_2_ (*P* = 0.095). The ventilation distribution of ROI2 was significantly decreased, and ROI3 and ROI4 were significantly increased, after 4 h of PP (Fig. 2 and S6). During PP, tidal impedance shifted significantly to the dorsal ROI. Once returned to supine positioning, ventral ROI tidal impedance was increased but still significantly less than before PP. Lung ultrasound scores in the anterior and posterior regions significantly decreased during PP. However, the changes in the lateral region were not significant (Fig. 3).

**Table 4:** Ventilatory variables, ECMO settings, arterial blood gas, electrical impedance tomography data, and lung ultrasonographic assessment along the first prone position cycle during ECMO for patients in the prone group

Variables	Supine before PP	During PP	End PP	Supine after PP	*P*
Ventilatory variables (*n* = 48)					
FiO_2_	0.39 ± 0.21	0.36 ± 0.17	0.33 ± 0.11	0.34 ± 0.08	0.161
PEEP (mmH_2_O)	12.8 ± 2.6	12.7 ± 2.7	12.7 ± 2.7	12.9 ± 2.9	0.737
Tidal volume (ml)	287 ± 79	283 ± 90	268 ± 87	281 ± 86	0.544
Tidal volume (ml/PBM)	4.6 ± 1.3	4.4 ± 1.4	4.2 ± 1.4	4.4 ± 1.5	0.356
Respiratory rate (breaths/min)	20.1 ± 6.2	17.8 ± 5.7	18.3 ± 6.6	19.7 ± 6.3	0.087
Peak airway pressure (cmH_2_O)	25.6 ± 2.9	25.4 ± 2.9	25.1 ± 3.0	24.7 ± 2.2	0.133
P_tp_ at end-inspiration (cmH_2_O)	14.2 ± 3.1	16.5 ± 4.3	16.6 ± 4.6	12.9 ± 3.1^#^	< 0.001
P_tp_ at end-expiration (cmH_2_O)	2.2 ± 3.0	4.4 ± 2.5	4.2 ± 3.0	2.5 ± 3.2	< 0.001
P_es_ at end-inspiration (cmH_2_O)	12.4 ± 1.2	9.9 ± 3.5	9.5 ± 3.2	12.8 ± 2.1	< 0.001
P_es_ at end-expiration (cmH_2_O)	10.6 ± 1.2	8.3 ± 2.6	8.5 ± 2.0	10.4 ± 2.2	< 0.001
ECMO settings (*n* = 50)					
ECMO blood flow (L/min)	3.96 ± 0.75	3.94 ± 0.69	3.93 ± 0.81	3.84 ± 0.97	0.863
Sweep gas flow (L/min)	4.0 ± 1.4	4.0 ± 1.3	4.0 ± 1.2	3.8 ± 1.3	0.419
FDO_2_	0.97 ± 0.06	0.98 ± 0.08	0.95 ± 0.11	0.97 ± 0.07	0.098
Arterial blood gas (*n* = 50)					
pH	7.45 ± 0.05	7.44 ± 0.05	7.44 ± 0.04	7.44 ± 0.05	0.714
PaO_2_ (mmHg)	70.3 ± 11.2	78.4 ± 12.4	78.7 ± 10.5	72.4 ± 10.2	< 0.001
PaCO_2_ (mmHg)	39.5 ± 8.3	40.0 ± 8.8	41.8 ± 6.3	41.7 ± 6.8	0.095
HCO_3_^−^ (mmol/L)	27.8 ± 3.3	26.7 ± 4.3	28.8 ± 4.1	28.6 ± 4.2	0.002
SaO_2_ (%)	95 ± 3	95 ± 2	96 ± 2	95 ± 2	0.699
Electrical impedance tomography (*n* = 47)					
ROI 1 of ventilation distribution (%)	9.5 ± 5.3	7.7 ± 6.5	7.8 ± 3.0	7.5 ± 3.3^#^	0.221
ROI 2 of ventilation distribution (%)	52.5 ± 9.7	37.6 ± 8.0	33.6 ± 6.9	42.4 ± 8.2*	< 0.001
ROI 3 of ventilation distribution (%)	30.4 ± 9.1	45.3 ± 10.3	47.9 ± 8.5	40.8 ± 8.1*	< 0.001
ROI 4 of ventilation distribution (%)	7.6 ± 3.6	9.3 ± 3.5	10.8 ± 3.2	9.3 ± 3.2^#^	< 0.001
Lung ultrasound scores (*n* = 48)					
Anterior region	12.2 ± 4.4	12.0 ± 4.9	11.3 ± 4.3	10.3 ± 3.9*	0.006
Lateral region	7.7 ± 2.4	7.3 ± 2.3	7.0 ± 2.1	7.4 ± 2.3	0.054
Posterior region	18.7 ± 5.3	17.0 ± 5.3	14.8 ± 5.0	15.8 ± 5.1*	< 0.001

ECMO venovenous extracorporeal membrane oxygenation, PP prone positioning, FiO_2_ the fraction of inspired oxygen, PEEP positive end-expiratory pressure, PBM predicted body weight, P_tp_ transpulmonary pressure, P_es_ esophageal pressure, PaO_2_ partial pressure of arterial oxygen, PaCO_2_ partial pressure of arterial carbon dioxide, HCO_3_^−^ bicarbonate, SaO_2_ arterial oxygen saturation, FDO_2_ fraction of delivered oxygen, ROI region of interest

^#^*P <* 0.05, * *P <* 0.01 compare with before PP.

### Propensity score matching

**Fig. 4:**
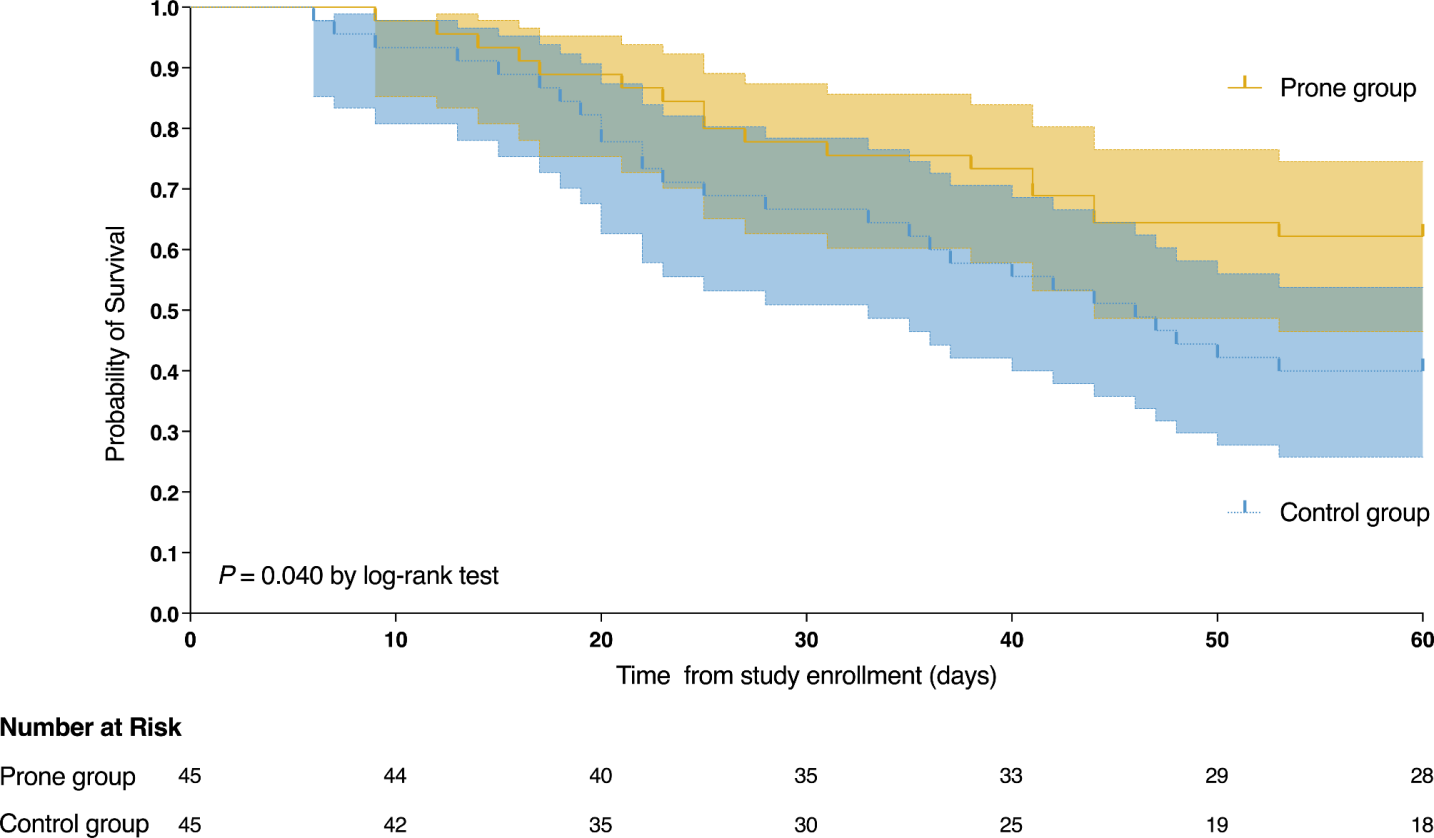
Probability of survival from day of initiating VV-ECMO to day 60 in matched groups of patients. VV-ECMO venovenous extracorporeal membrane oxygenation

Propensity score matching identified 90 patients, 45 in the prone and 45 in the supine group. Characteristics of the matched populations are displayed in Table S3. In this matched sample, the prone group had a higher proportion of patients successfully weaned from VV-ECMO (29 of 45 vs. 19 of 45, *P* = 0.035) and lower 60-day mortality (17 of 45 vs. 27 of 45, *P* = 0.035) than the supine group (Table 5). Comparison of the two survival curves showed the same significant difference (*P* = 0.040) (Fig. 4).

**Table 5:** Outcomes after propensity score matching analysis

Outcome	All patients (*n* = 90)	Prone group (*n* = 45)	Supine group (*n* = 45)	*P*
Primary end point				
Proportion of patients successfully weaned from VV-ECMO no. (%)	48 (53.3)	29 (64.4)	19 (42.2)	0.035
Secondary end point				
60-day mortality no. (%)	44 (48.9)	17 (37.8)	27 (60.0)	0.035
Other end points				
Successfully weaned from VV-ECMO for 30 days	46 (51.1)	28 (62.2)	18 (40.0)	0.035
ICU length of stay (days)	30 (20–45)	27 (19–42)	35 (21–51)	0.217
Hospital length of stay (days)	36 (25–50)	32 (27–48)	37 (23–55)	0.787
ECMO duration (days)	14 (8–20)	11 (8–18)	15 (9–24)	0.040
Ventilator-free days at day 60 (days)	0 (0–37)	26 (0–43)	0 (0–32)	0.047
CRRT no. (%)	45 (50.0)	23 (51.1)	22 (48.9)	0.833
Tracheostomy no. (%)	26 (28.9)	16 (35.6)	10 (22.2)	0.163

VV-ECMO venovenous extracorporeal membrane oxygenation, ICU intensive care unit, CRRT continuous renal replacement therapy, CRBSI catheter-related blood stream infection, VAP ventilator associated pneumonia

## Discussion

To our knowledge, this study is the first prospective observational study to investigate the approach of reapplying PP within 24 h after initiation of VV-ECMO in patients who underwent PP as rescue therapy before VV-ECMO. After PS matching, early use of PP after VV-ECMO was significantly associated with a higher proportion of patients successfully weaned from VV-ECMO and lower 60-day mortality. The EIT and lung ultrasound monitor showed a ventral-to-dorsal shift of tidal volume distribution and increased lung recruitment. Beyond the better outcome, PP during VV-ECMO appears safe, with no fatal complications.

The PROSEVA trial confirmed that PP for moderate to severe ARDS patients could reduce 28- and 90-day mortality [1]. A previous study showed that the more severe the ARDS, the greater the PP benefit [24]. Therefore, it is reasonable to use PP in patients receiving VV-ECMO [25]. However, PP was used infrequently after initiation of VV-ECMO, and several reasons may explain why. First, patients received PP before VV-ECMO, but perhaps no oxygenation improvement was observed, and there was a potential increased risk of complications during PP. Second, PP on ECMO is a resource-intensive task to implement safely. Staff physicians and nurses overwhelmed by the burden of the daily workload may not have no time to perform PP. Third, the impact on patient mortality remains uncertain.

In addition to numerous retrospective and observational studies, two large-scale meta-analyses have assessed the effect of PP in patients with severe ARDS receiving VV-ECMO. Unfortunately, they have inconsistent outcomes. The first study showed that use of PP in ARDS patients receiving VV-ECMO was associated with a significant improvement in 28-day survival (74 vs. 58%, *P* < 0.001) [10]. The second study analyzed the individual data of 889 patients and did not find that PP during VV-ECMO reduces ICU mortality (HR, 0.67 95%; CI: 0.42–1.06) [22].

The recently published PRONECMO study indicated that routine PP during VV-ECMO does not facilitate earlier weaning from ECMO or improve outcomes, findings that contrast with our results [17]. Notably, 94% of patients in the PRONECMO study had COVID-19-related ARDS, as revealed by the findings. In comparison, our study encompassed only 8% of patients with COVID-19-related ARDS, indicating a notable difference in the etiology of ARDS compared to the PRONECMO study. A previous study has reported that there may be distinct physiological responses to PP during VV-ECMO between patients with COVID-19-related ARDS and those with non-COVID-19-related ARDS. Furthermore, a recent study also failed to demonstrate the effectiveness of PP in COVID-19 patients treated with VV-ECMO [26]. Therefore, as mentioned in the PRONECMO study, the generalizability of its findings to individuals with severe non-COVID-19-related ARDS may be limited.

The initial reason for PP in ARDS patients was to alleviate severe hypoxemia, as it is an efficient way to improve oxygenation in most patients [27]. This research enrolled patients who had undergone PP before VV-ECMO. These patients were categorized as non-responders to PP in terms of oxygenation, which led to the initiation of VV-ECMO therapy. Should we no longer perform PP after VV-ECMO therapy? The relevance of this question appears low. PP benefits are independent of the oxygenation response and may be more related to less ventilator-induced lung injury (VILI), which is associated with higher rates of successful weaning from VV-ECMO [28]. In our study, a higher proportion of patients successfully weaned from VV-ECMO and alive was found in the prone group compared with a propensity-matched cohort of supine patients.

We believe that PP has temporal properties. Its potential benefit is likely affected by the timing of initiation and the duration of PP. In this study, we used PP as in the PROSEVA trial to ensure PP’s effectiveness during VV-ECMO [1]. Patients were placed in PP less than 24 h after VV-ECMO initiation for at least 16 consecutive hours. In addition, each patient underwent more than five sessions of PP during VV-ECMO unless stopping criteria were met.

EIT monitoring revealed PP-induced ventral-to-dorsal ventilation distribution changes in our study. The dorsal fraction of ventilation reached 60% by the end of PP. Perier et al. confirmed that PP did not change predominantly dorsal pulmonary perfusion [29]. Consequently, ventilation/perfusion matching improved after PP. Lastly, PaO_2_ improved without enhancement of blood flow on ECMO or excess mechanical ventilation. In the process, a slight decrease in tidal volume and respiratory rate underwent PP, which led to a subsequent increase in PaCO_2_ levels by 2mmHg. Lung ultrasound scores can detect alterations in lung aeration across position changes [30]. After extended PP, the anterior and posterior regions of the lung were recruited, leading to a decrease in P_tp_ at end-inspiration. Thus, PP might lead to a more homogeneous distribution of mechanical forces and enhance outcomes [31].

A recent study did not demonstrate any advantageous effects of biotrauma associated with an ultraprotective ventilation strategy, which included a P_tp_-guided ventilation approach combined with early PP during VV-ECMO support [32]. The utilization of P_tp_-guided ventilation approach to optimize PEEP can limit atelectrauma and minimize the risk of lung overdistention [19]. Both groups of patients were ventilated ≤ 4 ml/kg of PBM, with a difference of 1 ml/kg of PBM between the groups. Although these results were statistically significant, they were unable to lead to a reduction in the biotrauma. The patients in the ultra-lung-protective group only underwent 1–2 sessions of PP, significantly fewer than the 5–6 sessions of PP in our study. Repeated PP was associated with a gradual decrease in the probability of death [12]. Therefore, the ultraprotective ventilation strategy did not improve patient prognosis, and it even had a negative impact on outcomes due to factors such as increased use of neuromuscular blockers.

In our study, 60-day mortality in the prone group was similar to that reported in the CESAR and EOLIA trials. However, the supine position group exhibited a higher 60-day mortality compared to previous studies [5, 6]. Two factors could account for this phenomenon. First, PP before ECMO was associated with a lower probability of 90-day ICU discharge alive [14]. Second, the mean values of PaO_2_/FiO_2_ in the CESAR and EOLIA trials were 76 mm Hg and 73 mm Hg, respectively. However, in our study, the median PaO_2_/FiO_2_ was 60 mm Hg, significantly lower than that of the previous two studies [5, 6]. This indicates that the patients in our study had more severe illness, which is associated with poorer outcomes.

Beyond being the first study that specifically explored the role of early reapplication of PP after VV-ECMO initiation in patients with severe ARDS, our study had several limitations. First, this is a single-center prospective study with a relatively small sample size. Further multicenter large-scale trials should be conducted to validate and expand upon these findings. Second, because treatment was not randomly allocated, PP use was based on the treating physicians’ clinical judgment. Despite applying a propensity score-matching analysis, we cannot exclude a selection bias in patients who underwent PP during VV-ECMO. Third, a P_tp_-guided ventilation strategy during VV-ECMO was used. Thus, our findings might not be generalizable to centers with different ventilatory approaches. Fourth, fluid balance and depth of sedation during PP might affect survival and respiratory mechanics, but its effect was not assessed. Fifth, it is important to note that the study only a limited number of COVID-19-related ARDS (8%) were included. Thus, our findings might not be generalizable to ARDS due to severe COVID-19. Last, ventilatory variables were recorded only during the first PP cycle after VV-ECMO support. Therefore, we were unable to assess any changes in these variables over time.

## Conclusions

In conclusion, our study demonstrates that patients with severe ARDS placed in the prone position before VV-ECMO initiation should continue PP after VV-ECMO support. This approach could increase the proportion of these patients successfully weaned from VV-ECMO and alive. Selection of patients who could most benefit from PP during VV-ECMO is urgently needed.

### Electronic supplementary material

Below is the link to the electronic supplementary material.

**Additional file 1**: 1. Online Methods Supplement. 1.1 Section S1: Procedures for performing prone positioning in VV-ECMO patients. 1.2 Section S2: Measurement of esophageal pressure. 2. Online Table 2.1 Table S1: Missing data. 2.2 Table S2: Univariate logistic analysis for patients successfully weaned from VV-ECMO. 2.3 Table S3: Demographic characteristics, pre-ECMO treatments, hemodynamic status, arterial blood gas and ventilatory variables after propensity score matching analysis. 3. Online Fig. 3.1 Figure S1: Healthcare workers’ position for performing prone positioning in VV-ECMO patients. 3.2 Figure S2: The surface anatomy of the lung lobes and lung ultrasound zones. 3.3 Figure S3: Dot plots of absolute standardized mean differences before and after propensity score matching. 3.4 Figure S4: Flow chart. 3.5 Figure S5: Probability of survival from the day of initiating VV-ECMO to day 60 in the prone and supine group. 3.6 Figure S6: Changes in ventilation distribution at four ventral-to-dorsal horizontal regions across the first PP session after VV-ECMO support.

**Additional file 2**

## Data Availability

The data are available from the corresponding author on reasonable request.

## Abbreviations

PP: Prone positioning
ARDS: Acute respiratory distress syndrome
COVID-19: Coronavirus disease 2019, VV-ECMO: venovenous extracorporeal membrane oxygenation
ICU: Intensive care unit
PBM: Predicted body weight
PEEP: Positive end-expiratory pressure
FiO_2_: Fraction of inspired oxygen
PaCO_2_: Partial pressure of arterial carbon dioxide
P_plat_: Plateau pressure
MAP: Mean arterial pressure
SpO_2_: Saturation of pulse oxygen
P_tp_: Transpulmonary pressure
P_peak_: Peak airway pressure
APTT: Activated partial thromboplastin time
RASS: Richmond Agitation and Sedation Scale
EIT: Electrical impedance tomography
ROI: Region of interest
CRRT: Renal replacement therapy
PS: Propensity score
SOFA: Sequential organ failure assessment
VILI: Ventilator-induced lung injury
