# Supplementary material for: Early reapplication of prone position during venovenous ECMO for acute respiratory distress syndrome: a prospective observational study and propensity-matched analysis
Source: Ann Intensive Care. 2024 Aug 20;14:127. doi: 10.1186/s13613-024-01365-4 (PMC11336129; doi:10.1186/s13613-024-01365-4)
Supplement: Supplementary file 1 — Additional file 1: 1. Online Methods Supplement. 1.1 Section S1: Procedures for performing prone positioning in VV-ECMO patients. 1.2 Section S2: Measurement of esophageal pressure. 2. Online Table 2.1 Table S1: Missing data. 2.2 Table S2: Univariate logistic analysis for patients successfully weaned from VV-ECMO. 2.3 Table S3: Demographic characteristics, pre-ECMO treatments, hemodynamic status, arterial blood gas and ventilatory variables after propensity score matching analysis. 3. Online Fig. 3.1 Figure S1: Healthcare workers’ position for performing prone positioning in VV-ECMO patients. 3.2 Figure S2: The surface anatomy of the lung lobes and lung ultrasound zones. 3.3 Figure S3: Dot plots of absolute standardized mean differences before and after propensity score matching. 3.4 Figure S4: Flow chart. 3.5 Figure S5: Probability of survival from the day of initiating VV-ECMO to day 60 in the prone and supine group. 3.6 Figure S6: Changes in ventilation distribution at four ventral-to-dorsal horizontal regions across the first PP session after VV-ECMO support. [file 13613_2024_1365_MOESM1_ESM.docx]

**ADDITIONAL FILE 1: SUPPLEMENTARY MATERIAL**

**Early reapplication of prone position during venovenous ECMO for acute respiratory distress syndrome: A prospective observational study and propensity-matched analysis**

Rui Wang, MD^1^; Xiao Tang, MD^1^; Xuyan Li, MD^1^; Ying Li, MD^1^; Yalan Liu, MD^1^; Ting Li, MD^1^; Yu Zhao, MD^1^; Li Wang, MD^1^; Haichao Li, MD^1^; Meng Li, MD^1^; Hu Li, MD^1^; Zhaohui Tong MD^1^ Bing Sun, MD^1^

**Author Affiliations:**

1. Department of Respiratory and Critical Care Medicine, Beijing Institute of Respiratory Medicine and Beijing Chao-Yang Hospital, Capital Medical University, No. 8 Gongren Tiyuchang Nanlu, Chaoyang District, Beijing 100020, China.

**Corresponding author:**

Bing Sun MD

Department of Respiratory and Critical Care Medicine, Beijing Institute of Respiratory Medicine and Beijing Chao-Yang Hospital, Capital Medical University, No. 8 Gongren Tiyuchang Nanlu, Chaoyang District, Beijing 100020, China.

Email: ricusunbing@126.com

**1. Online Methods Supplement**

**1.1 Section S1: Procedures for performing prone positioning in VV-ECMO patients**

The prone position maneuver in patients treated with venovenous extracorporeal membrane oxygenation (VV-ECMO) usually requires six healthcare workers to participate. One respiratory therapist should be dedicated to managing the head of the patient, including protecting the ECMO jugular cannula and endotracheal tube. Two nurses at each side of the bed turn the patient. Moreover, one of the nurses takes care of the femoral ECMO cannula. During the prone position maneuver, one physician pays full attention to the ECMO flow and the integrity and potential displacement of the ECMO lines.

The direction of the turning is critical. Turning should prioritize the reinjection line of VV-ECMO, leaving them on the top during the turning. It should also be noted that pillows are necessary to avoid compression of the femoral cannulas and to facilitate the correct assessment of the insertion site to detect any bleeding.

**1.2 Section S2: Measurement of esophageal pressure**

We used the AVEA® ventilator (CareFusion, U.S.A) and AVEA® SmartCath® Esophageal Pressure Monitoring Tube Sets (CareFusion, U.S.A) to measure esophageal pressure (Pes). And this tube could also be used as gastric tube. It was simple and easy to place the esophageal pressure monitoring tube, which needed no additional invasive operation. This special tube required an additional cost, but the patients didn't need to pay for it during clinical trials.

After the leak test in vitro, the esophageal balloon was ready for placement in the patient. We located the approximate depth of placement by measuring the distance from the tip of the nose to the bottom of the earlobe and from the earlobe to the distal tip of the xiphoid process. After balloon placement using this method, the esophageal pressure waveform correlates to the airway pressure in that it becomes positive during a positive-pressure breath and negative during a spontaneous breath. The esophageal tracing may show small cardiac oscillations that represent cardiac activity. We confirmed the appropriate balloon location using an occlusion technique. This required that the airway be occluded and the change in airway (∆Paw) and esophageal (∆Pes) pressure be almost identical (∆Pes/∆Paw ratio, 0.8-1.2). The ventilator automatically evacuates and refills the balloon every 30 minutes to ensure the accuracy of monitored values.

**2. Online Tables Supplement**

2.1 Table S1: Missing data

| Variable of first prone position cycle during ECMO for patients in the prone group | Number of patients with missing data |
| --- | --- |
| Ventilatory variables |  |
| P_tp_ at end-inspiration | 2 |
| P_tp_ at end-expiration |  |
| P_es_ at end-inspiration |  |
| P_es_ at end-expiration |  |
| Electrical impedance tomography |  |
| ROI 1 of ventilation distribution | 3 |
| ROI 2 of ventilation distribution |  |
| ROI 3 of ventilation distribution |  |
| ROI 4 of ventilation distribution |  |
| Lung ultrasound scores | 2 |
| Anterior region |  |
| Posterior region |  |

ECMO venovenous extracorporeal membrane oxygenation, P_tp_ transpulmonary pressure, P_es_ esophageal pressure, ROI region of interest

2.2 Table S2: Univariate logistic analysis for patients successfully weaned from VV-ECMO

| Variable | β Coefficient | Standard Error | Odds ratios (95% *CI*) | *P* |
| --- | --- | --- | --- | --- |
| Age | -0.177 | 0.027 | 0.837 (0.795 – 0.882) | < 0.001 |
| Body mass index (kg/m^2^) | -0.002 | 0.057 | 0.998 (0.893 – 1.115) | 0.970 |
| SOFA score | -0.014 | 0.056 | 0.986 (0.883 – 1.102) | 0.808 |
| Immunocompromised (reference no) | -1.278 | 0.438 | 0.279 (0.118 – 0.658) | 0.004 |
| Days of MV before ECMO (days) | -0.011 | 0.003 | 0.989 (0.983 – 0.995) | < 0.001 |
| Prone positioning |  |  |  |  |
| Duration of PP (hours/day) | 0.137 | 0.047 | 1.147 (1.046 - 1.258) | 0.004 |
| Number of PP (days) | -0.415 | 0.089 | 0.660 (0.554 – 0.787) | < 0.001 |
| PaO_2_:FiO_2_ ratio before ECMO (mmHg) | -0.009 | 0.012 | 0.991 (0.967 – 1.015) | 0.447 |

VV-ECMO venovenous extracorporeal membrane oxygenation, SOFA sequential organ failure assessment, MV mechanical ventilation, PP, prone position, PaO_2_:FiO_2_ ratio of the partial pressure of arterial oxygen to the fraction of inspired oxygen

2.3 Table S3: Demographic characteristics, pre-ECMO treatments, hemodynamic status, arterial blood gas and ventilatory variables after propensity score matching analysis

| Characteristic | All patients  (n = 90) | Prone group  (n = 45) | Supine group  (n = 45) | Absolute standardized mean difference |
| --- | --- | --- | --- | --- |
| Age (years) | 58.6 ± 14.1 | 57.8 ± 13.8 | 59.2 ± 14.6 | 0.099 |
| Male, no. (%) | 65 (72.2) | 33 (73.3) | 32 (71.1) | 0.049 |
| Body mass index (kg/m^2^) | 25.0 (22.9 – 26.1) | 25.0 (22.9 – 26.6) | 24.9 (22.4 – 25.7) | 0.083 |
| Pulmonary pathogen spectrum, no. (%) |  |  |  |  |
| Bacterial | 22 (24.4) | 11 (24.4) | 11 (24.4) | 0.000 |
| Viral | 51 (56.7) | 26 (57.8) | 25 (55.6) | 0.044 |
| COVID-19 pneumonia | 6 (6.7) | 3 (6.7) | 3 (6.7) | 0.000 |
| Fungal | 2 (2.2) | 1 (2.2) | 1 (2.2) | 0.000 |
| Pneumocystis jiroveci | 15 (16.7) | 7 (15.6) | 8 (17.8) | 0.059 |
| Comorbidity, no. (%) |  |  |  |  |
| Immunocompromised | 19 (21.1) | 10 (22.2) | 9 (20.0) | 0.054 |
| Coronary artery disease | 13 (14.4) | 6 (13.3) | 7 (15.2) | 0.054 |
| Hypertension | 42 (46.7) | 20 (44.4) | 22 (48.9) | 0.090 |
| Diabetes mellitus | 16 (17.8) | 8 (17.8) | 8 (17.8) | 0.000 |
| Chronic renal insufficiency | 7 (7.8) | 4 (8.9) | 3 (6.7) | 0.082 |
| Pre-Scores |  |  |  |  |
| Murray score | 3.50 (3.50 - 3.75) | 3.50 (3.50 - 3.75) | 3.50 (3.50 - 3.75) | 0.036 |
| SOFA score | 12 (10 - 14) | 12 (11 - 14) | 12 (10 - 14) | 0.039 |
| APACHE II score | 18 (14 - 22) | 17 (14 - 22) | 18 (13 - 22) | 0.082 |
| RESP score | 2 (0 - 3) | 2 (0 - 4) | 2 (1 - 3) | 0.057 |
| Pre-ECMO variables |  |  |  |  |
| Duration of MV before ECMO (hours) | 33 (18 - 78) | 44 (24 - 70) | 7 (25 - 102) | 0.068 |
| Rescue therapy, no. (%) |  |  |  |  |
| Corticosteroids | 21 (23.3) | 11 (24.4) | 10 (22.2) | 0.052 |
| Prone positioning | 90 (100.0) | 45 (100.0) | 45 (100.0) | 0.000 |
| Duration of PP (hours/day) | 15 (13 - 17) | 16 (14 - 17) | 15 (13 - 17) | 0.074 |
| Number of PP sessions | 2 (1 - 3) | 2 (1 - 3) | 1 (1 - 4) | 0.062 |
| Lung recruitment maneuvers | 40 (44.4) | 21 (46.7) | 19 (42.2) | 0.091 |
| Neuromuscular blockade | 11(12.2) | 6 (13.3) | 5 (11.1) | 0.067 |
| HFOV | 5 (5.6) | 2 (4.4) | 3 (6.7) | 0.100 |
| Inhaled nitric oxide | 7 (7.8) | 3 (6.7) | 4 (8.9) | 0.082 |
| Hemodynamic status |  |  |  |  |
| Vasopressor, no. (%) | 64 (71.1) | 33 (73.3) | 31 (68.9) | 0.097 |
| Lactate (mmol/L) | 1.9 (1.4 – 2.7) | 1.7 (1.2 – 2.9) | 1.9 (1.6 – 2.4) | 0.025 |
| Heart rate (beats/min) | 104.8 ± 24.3 | 103.7 ± 26.5 | 105.9 ± 22.1 | 0.090 |
| Mean arterial pressure (mmHg) | 76.6 ± 12.7 | 77.0 ± 12.5 | 76.1 ± 13.0 | 0.070 |
| Arterial blood gas |  |  |  |  |
| pH | 7.36 (7.28 – 7.42) | 7.36 (7.30 – 7.43) | 7.36 (7.27 – 7.42) | 0.059 |
| PaO_2_ (mmHg) | 59.1 ± 11.3 | 59.4 ± 10.7 | 58.7 ± 11.9 | 0.064 |
| PaCO_2_ (mmHg) | 47.3 ± 12.2 | 47.2 ± 12.1 | 47.5 ± 12.4 | 0.029 |
| HCO_3_^-^ (mmol/L) | 25.3 ± 5.5 | 25.1 ± 5.8 | 25.5 ± 5.2 | 0.076 |
| SaO_2_ (%) | 89 (85 - 92) | 89 (86 - 91) | 88 (84 - 92) | 0.081 |
| PaO_2_:FiO_2_ ratio (mmHg) | 59.5 ± 11.7 | 59.6 ± 10.7 | 59.4 ± 12.8 | 0.017 |
| Ventilatory variables |  |  |  |  |
| PEEP (mmH_2_O) | 14.5 ± 3.6 | 14.7 ± 2.9 | 14.4 ± 4.2 | 0.086 |
| Tidal volume (ml) | 399 ± 57 | 409 ± 59 | 398 ± 55 | 0.062 |
| Tidal volume (ml/PBM) | 5.95 ± 0.71 | 5.98 ± 0.93 | 5.92 ± 0.40 | 0.089 |
| Respiratory rate (breaths/min) | 28.5 ± 5.4 | 28.6 ± 4.0 | 28.4 ± 6.5 | 0.041 |
| Plateau pressure (cmH_2_O) | 28.4 ± 3.9 | 28.4 ± 4.0 | 28.3 ± 3.9 | 0.013 |
| Peak airway pressure (cmH_2_O) | 32.1 ± 3.5 | 32.0 ± 3.1 | 32.2 ± 3.8 | 0.063 |
| Driving pressure (cmH_2_O) | 13.8 ± 3.5 | 13.7 ± 4.2 | 14.0 ± 2.8 | 0.076 |
| Compliance (ml/cmH_2_O) | 26.0 ± 7.2 | 26.1 ± 7.0 | 25.8 ± 7.5 | 0.033 |

COVID-19 coronavirus disease 2019, SOFA sequential organ failure assessment, APACHE II Acute Physiology and Chronic Health Evaluation II, ECMO extracorporeal membrane oxygenation, RESP Respiratory ECMO Survival Prediction, MV mechanical ventilation, HFOV high frequency oscillatory ventilation, PaO_2_ partial pressure of arterial oxygen, PaCO_2_ partial pressure of arterial carbon dioxide, HCO_3_^-^ bicarbonate, SaO_2_ arterial oxygen saturation, FiO_2_ the fraction of inspired oxygen, PaO_2_:FiO_2_ ratio of the partial pressure of arterial oxygen to the fraction of inspired oxygen, PEEP positive end-expiratory pressure, PBM predicted body weight

**3. Online Figures Supplement**

3.1 Figure S1: Healthcare workers' position for performing prone positioning in VV-ECMO patients


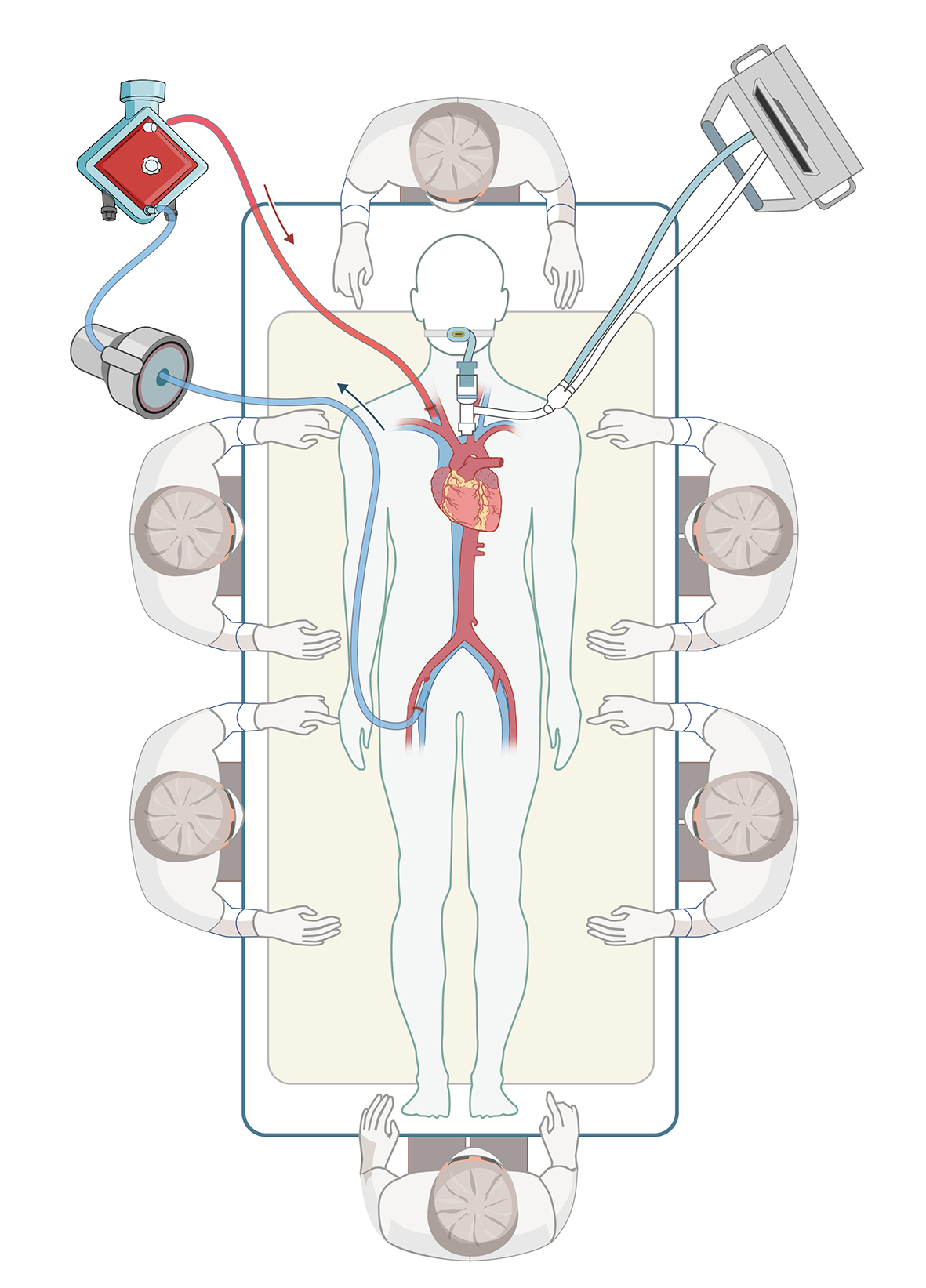


3.2 Figure S2: The surface anatomy of the lung lobes and lung ultrasound zones


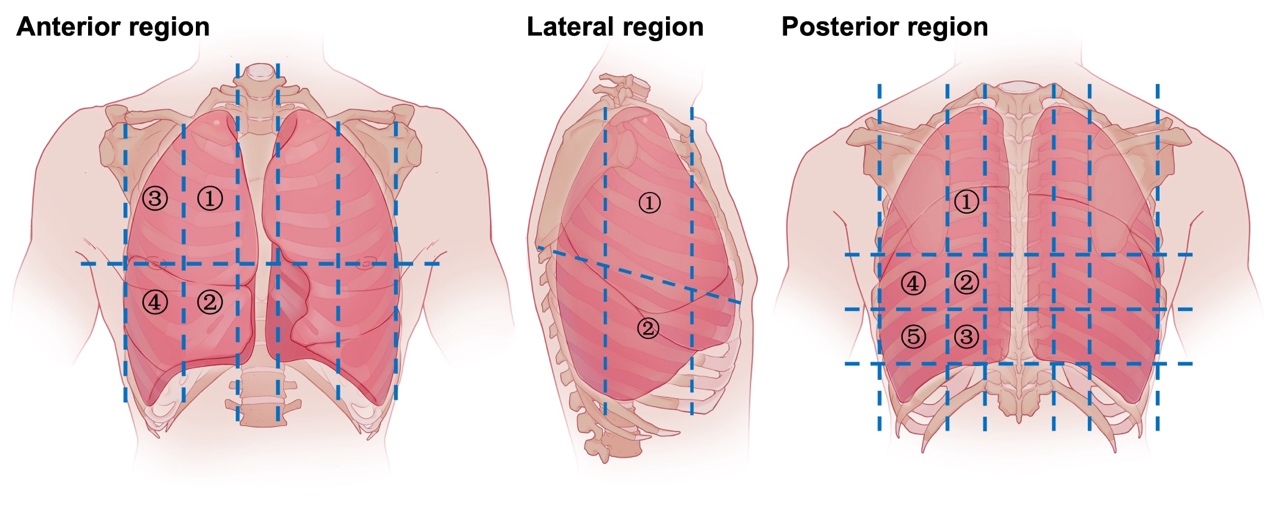


Each lung was divided into anterior, lateral, and posterior regions by parasternal, anterior axillary line, posterior axillary line, and paravertebral lines The anterior region was equally divided into four areas by the clavicular midline and the horizontal line. The lateral region was divided into the upper and lower zone by the horizontal line. The parascapular line divided the posterior region into two unequal areas, then every area was divided into three areas by two horizontal lines to get five examination areas (the scapula covers one was ruled out).

3.3 Figure S3: Dot plots of absolute standardized mean differences before and after propensity score matching


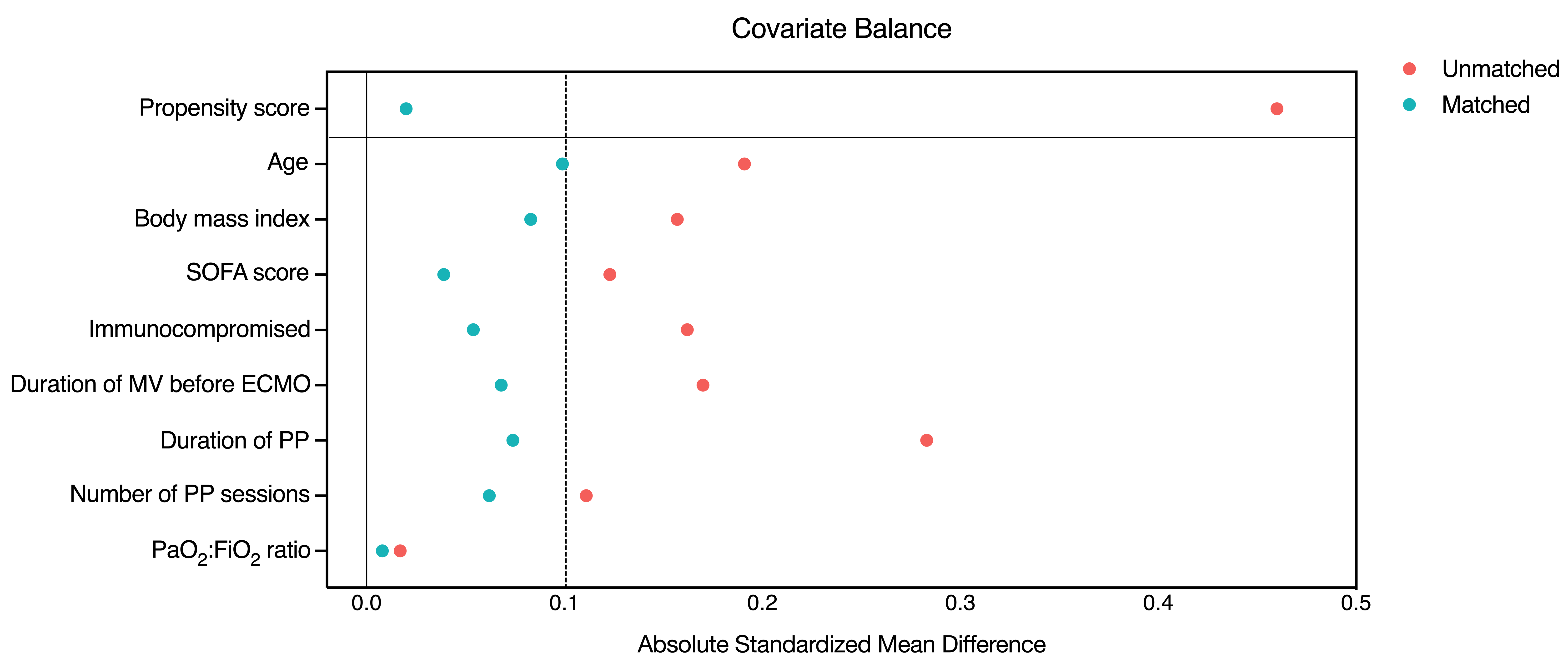


SOFA sequential organ failure assessment, MV mechanical ventilation, ECMO extracorporeal membrane oxygenation, PaO_2_:FiO_2_ ratio of the partial pressure of arterial oxygen to the fraction of inspired oxygen

3.4 Figure S4: Flow chart


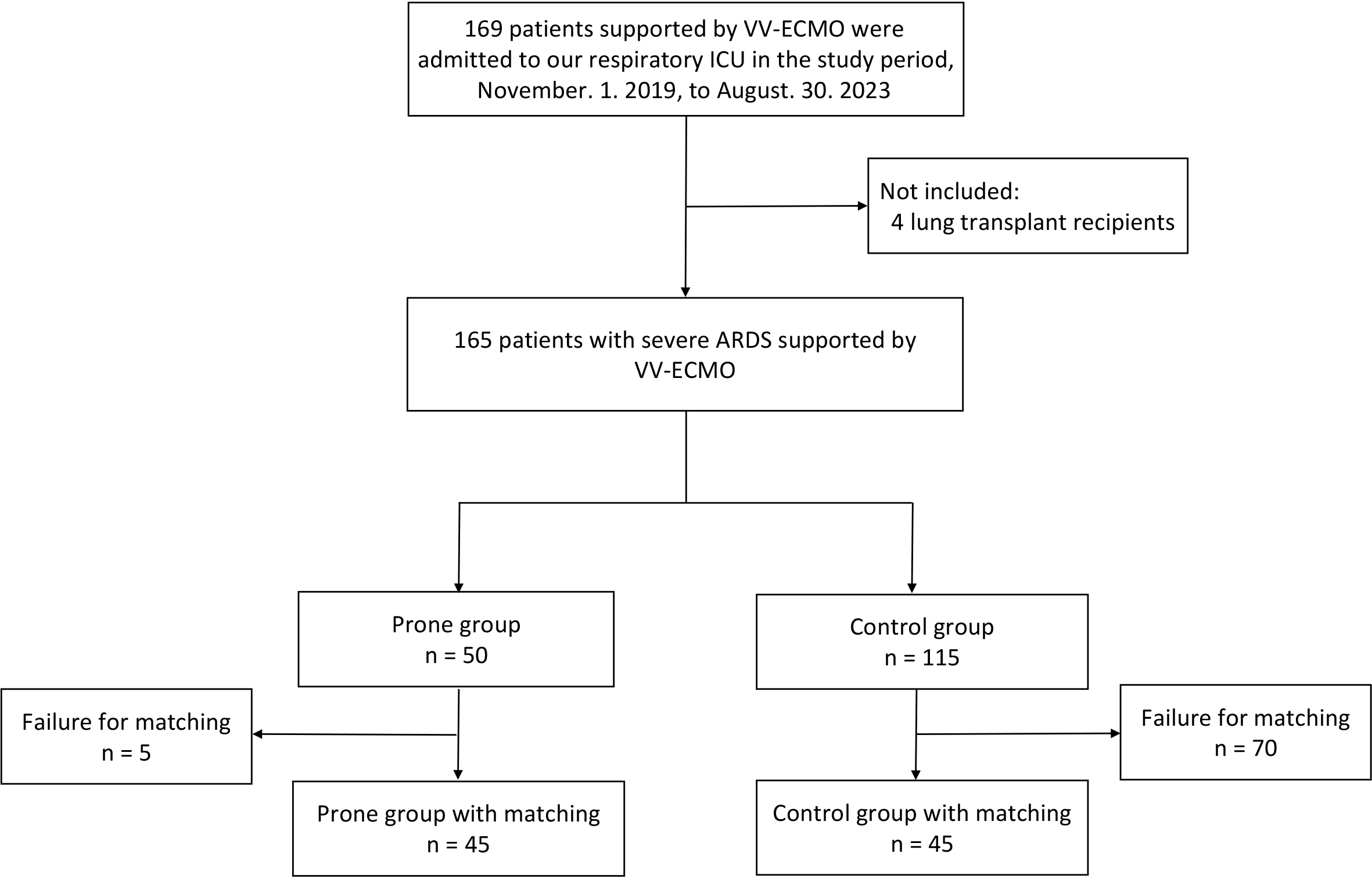


VV-ECMO venovenous extracorporeal membrane oxygenation, ICU intensive care unit, ARDS acute respiratory distress syndrome.

3.5 Figure S5: Probability of survival from the day of initiating VV-ECMO to day 60 in the prone and supine group


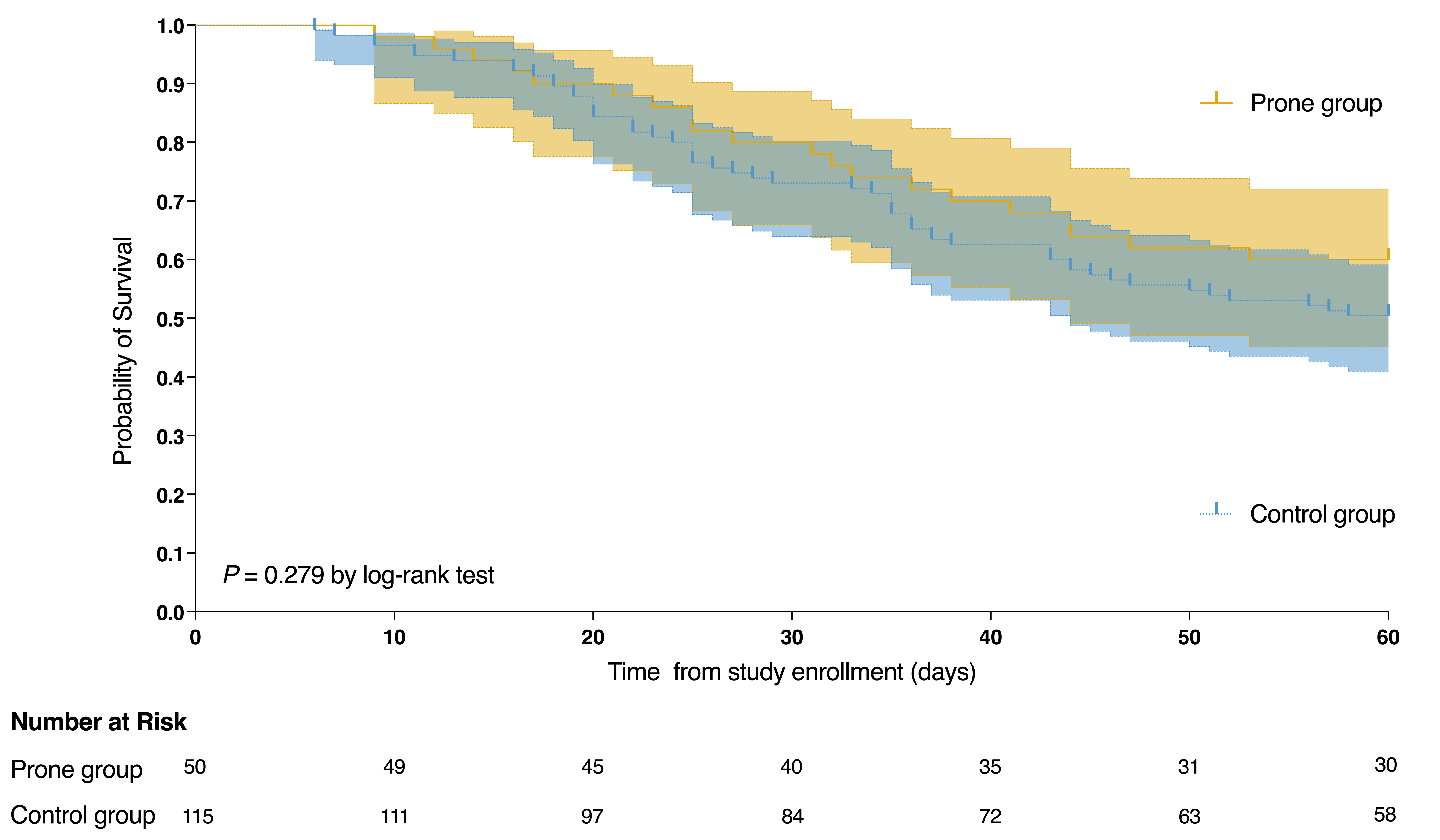


VV-ECMO venovenous extracorporeal membrane oxygenation.

3.6 Figure S6: Changes in ventilation distribution at four ventral-to-dorsal horizontal regions across the first PP session after VV-ECMO support


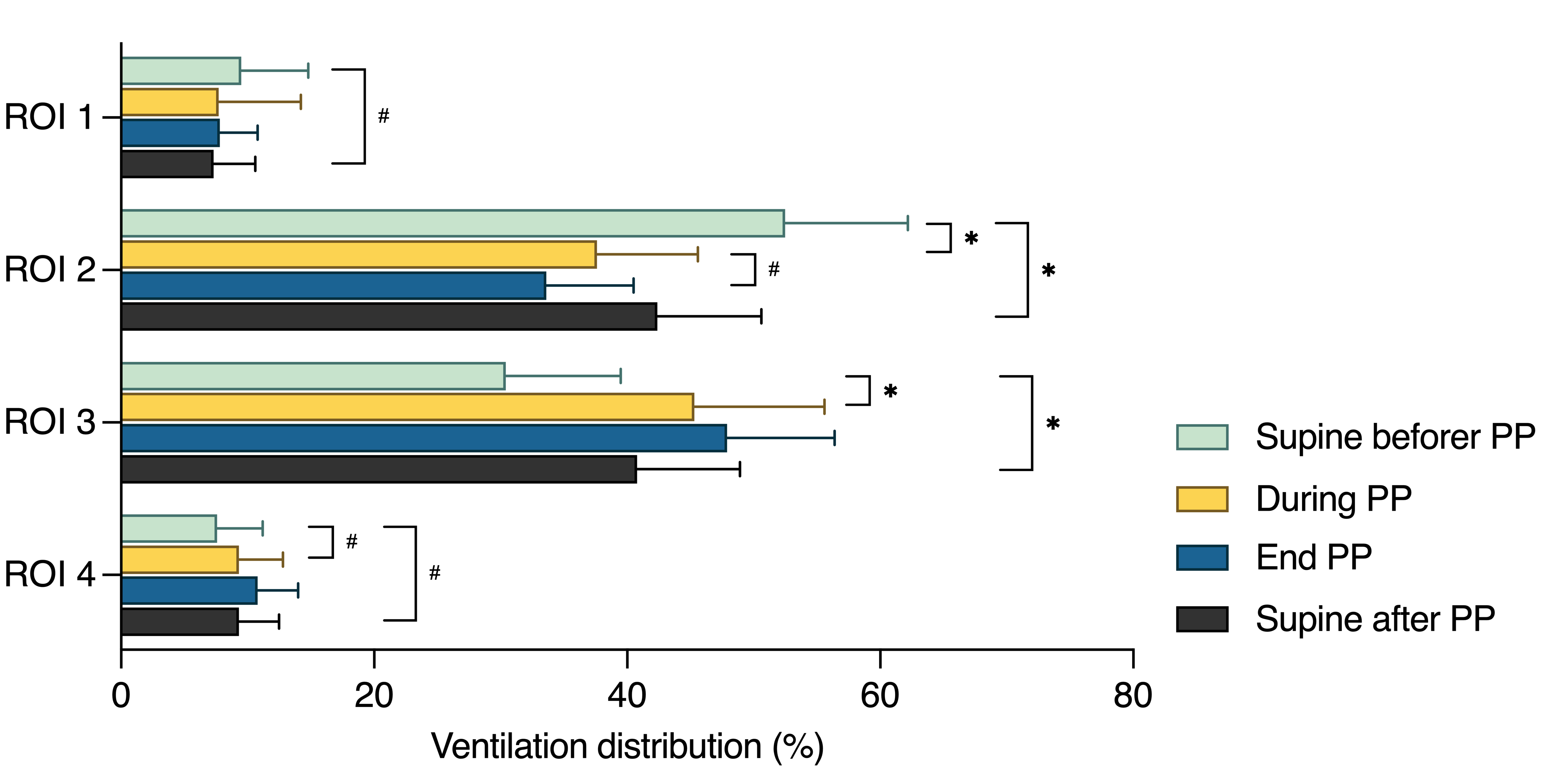
 PP prone position, VV-ECMO venovenous extracorporeal membrane oxygenation, ROI regions of interest.

^#^ *P <*0.05, * *P <*0.01.
